# Fear of COVID-19 among cancer patients in Henan Province, Central China: causes, results, and coping factors

**DOI:** 10.3389/fpsyg.2023.1122894

**Published:** 2023-06-16

**Authors:** Yiqing Mao, Wenjie Ma, Dingding Kang, Yudong Miao, Hang Fu, Bowen Zhang, Jiangong Zhang, Jian Wu

**Affiliations:** ^1^Affiliated Cancer Hospital of Zhengzhou University, Zhengzhou, China; ^2^College of Public Health, Zhengzhou University, Zhengzhou, China; ^3^Institute for Hospital Management of Henan Province, Zhengzhou, China; ^4^The First Affiliated Hospital of Zhengzhou University, Zhengzhou, China

**Keywords:** COVID-19 fear level, cancer patients, cause factors, result factors, coping factors, Central China

## Abstract

**Objectives:**

Cancer patients exhibit fear of COVID-19, which could lead to serious consequences. However, minimal information is available about the effect of the COVID-19 pandemic on the mental health of cancer patients. Therefore, this study aims to examine the fear level of COVID-19 among cancer patients in Henan Province, Central China and to identify its causes, results, and coping factors.

**Methods:**

An online survey was conducted among 1,067 cancer patients. The participants reported their individual fear level of COVID-19, risk of COVID-19 infection, risk of death from COVID-19, COVID-19 vaccination concerns, influence level of COVID-19 pandemic on their disease treatment, loneliness due to COVID-19, economic burden from COVID-19, quality of life, safety behavior, information regarding COVID-19 vaccination, psychological guidance, physical activities, and demographic characteristics. Chi-square and cumulative logistic regression were used to determine the predictors of COVID-19 fear level.

**Results:**

This study indicates that cancer patients report moderate fear level of COVID-19 in Central China (66.9%). The six cause factors (risk of COVID-19 infection, risk of death from COVID-19, COVID-19 vaccination concerns, influence level of COVID-19 pandemic on disease treatment, loneliness due to COVID-19, and economic burden from COVID-19) were positively associated with COVID-19 fear level. Three coping factors (information regarding COVID-19 vaccination, psychological guidance, and physical activities) were negatively associated with COVID-19 fear level. COVID-19 fear level was negatively associated with quality of life and positively associated with safety behavior.

**Conclusion:**

Our results suggest that governments should improve access to personalized vaccine counseling and psychological guidance by undertaking the responsibility of patients’ attending physicians and increasing publicity. Physical activities should be included in the treatment program to help cancer patients better recover their physical and mental health.

## Introduction

To date, COVID-19 has been spread around the world for 3 years. As a vulnerable group during the COVID-19 epidemic, cancer patients have suffered from a high level of fear associated with COVID-19 (Anuk, 2022). Previous research has shown that more than 90% of cancer patients exhibit moderate or severe fear of COVID-19 (Guven et al., 2020b). Fear of COVID-19 may lead to serious consequences, such as a weakened immune system, treatment compliance disorder, and worsening prognosis, among cancer patients (Erdogan et al., 2022). Therefore, under the realistic background of COVID-19’s universalization, we must give attention to public health problems that result from the fear of COVID-19 among cancer patients. Although the number of literature on COVID-19 has increased rapidly, and physical and psychological aspects have been considered, minimal information is available about the effect of the COVID-19 pandemic on the mental health of vulnerable patient groups, such as cancer patients (Musche et al., 2020). What are the causes of their fear? What are the consequences of their state of fear? How should this phenomenon be addressed? Existing research lacks systematic answers to these questions. Accordingly, the current study aims to assess the COVID-19 fear level of Chinese cancer patients and then identify the causes, results, and coping factors of fear of COVID-19.

## Causes of fear of COVID-19

At present, scholars have emphasized that an individual’s fear of COVID-19 primarily stems from the harm caused by COVID-19 to one’s health and the obstacles it poses to socioeconomic development (Mertens et al., 2021). In terms of health factors, cancer patients faced an increased risk of COVID-19 morbidity and mortality compared with the normal population. Previous research has shown that cancer patients with who become infected with COVID-19 have a twofold risk of death (Bernard et al., 2021) and a threefold risk of developing serious complications from COVID-19 compared with those without cancer (Wang et al., 2020). Hence, researchers believe that cancer patients perceive a higher possibility of COVID-19 infection and mortality, increasing their fear level of COVID-19 (Guven et al., 2020b; Yahaghi et al., 2021). Concerns regarding COVID-19 vaccine are also widespread among cancer patients because of the underrepresentation of such patients in the COVID-19 vaccine trial. Moreover, some subgroups of cancer have received different recommendations for vaccination and timing (Fendler et al., 2022; Vanderpool et al., 2022). In Poland, only 40.4% of cancer patients believe that the vaccine is effective for them (Kufel-Grabowska et al., 2021). In the United States, 39% of cancer patients are worried about the vaccine (van der Veldt et al., 2021). Previous research has indicated that concern about the protective effect of the COVID-19 vaccine can increase the fear of COVID-19 among cancer patients (Can and Kurtulus, 2021). In addition to the direct consequences of the pandemic, patients cannot go to hospitals because they worry over becoming infected with COVID-19, along with the prevention and control policies for COVID-19. However, the decline in cancer screening and the cessation of treatment can increase the COVID-19 fear level of cancer patients (Guven et al., 2020b; Anuk, 2022). In terms of socioeconomic factors, cancer patients may suffer from severe loneliness because of the strict social interaction restrictions and isolation policies imposed during the COVID-19 pandemic, which may aggravate the fear of COVID-19 (Humphrey et al., 2022). For cancer patients with economic burden, the decline of income caused by COVID-19 is another reason why they fear COVID-19 (Kirby et al., 2022).

Therefore, we assume that six cause factors are positively associated with COVID-19 fear level, as follows: risk of COVID-19 infection, risk of death from COVID-19, concerns regarding COVID-19 vaccination, influence level of COVID-19 outbreak on disease treatment, loneliness, and economic burden from COVID-19.

## Results of fear of COVID-19

For most people, fear of COVID-19 triggers an anxiety response, which is either adaptive (i.e., plays an incentive role in behavior change) or poorly adaptive (i.e., overall quality of life deteriorates) (Korajlija and Jokic-Begic, 2020). Previous research has shown that fear affects the overall quality of life, and this finding has been confirmed in many studies (Naghizadeh and Mirghafourvand, 2021; Demirbas and Kutlu, 2022). In addition to the adverse effects of fear, some studies have indicated that an individual’s fear exerts positive effects on safety behavior. Safety behavior, such as handwashing and physical distancing, is adherent and actively prescribed by government authorities during the COVID-19 pandemic to curtail infection rates. However, such behavior requires individuals to incur an immediate cost for the sake of society (Weismuller et al., 2021). Emotion–motivation models predict that negative emotions will lead to avoidance behavior, i.e., safety behavior during the COVID-19 pandemic (Hein et al., 2022). Therefore, numerous studies have demonstrated that safety behavior is associated with fear (Parlapani et al., 2020; Fink et al., 2021). Some scholars have even proposed that fear is one of the most powerful factors that affects safety behavior (Demirtas-Madran, 2021). Negative effects related to COVID-19, such as fear, have been predicted to prompt people to avoid potential sources of infection, improving compliance with safety behavior related to COVID-19.

Therefore, the current study assumes that fear of COVID-19 will lead to two results: quality of life deterioration and adoption of safety behavior. COVID-19 fear level is negatively associated with quality of life and positively associated with safety behavior.

## Coping with fear of COVID-19

Previous studies have indicated that coping methods for COVID-19 includes those at the individual and social levels. Firstly, the contribution of the health system to reducing the fear level of COVID-19 is the most important. Vaccination is recognized worldwide as the most important protective measure for reducing the infection and mortality rates of COVID-19. Previous studies have shown that vaccination and vaccine cognition can be associated with an individual’s fear level of COVID-19 (Can and Kurtulus, 2021; Gao et al., 2022). Given the different recommendations on vaccination and timing for some subgroups of cancer, cancer patients are more cautious about COVID-19 vaccination and tend to seek professional advice than the general population. Therefore, we believe that professional vaccination advice can help cancer patients improve their confidence in the effectivity of the COVID-19 vaccine, reducing their COVID-19 fear level. Meanwhile, many studies have proposed that psychological guidance should be strengthened to reduce an individual’s fear of COVID-19 (Guo et al., 2022); however, only a few studies have confirmed the value of psychological counseling. Second, in terms of individual-level coping measures, a previous research examined associations between physical activities and mental health during the COVID-19 lockdown in the United Kingdom (Wright et al., 2021) and Arab countries (Alsalhe et al., 2020). The results found that physical activities during the COVID-19 pandemic can counteract the negative effects of fear on mental health. This study suggested that physical activities may play a positive role in reducing the fear of COVID-19.

Therefore, we assume that the coping factors for fear of COVID-19 include the following: information about COVID-19 vaccination, psychological guidance, and physical activities. These factors are negatively associated with COVID-19 fear level.

## Characteristics of fear of COVID-19

In addition to causes, results and coping factors of fear level of COVID-19, previous studies have shown that some characteristics factors were associated with COVID-19 fear level. For example, individual with female (Broche-Perez et al., 2020), young (Levy and Cohen-Louck, 2021), unemployed (Levy and Cohen-Louck, 2021), low income (Argabright et al., 2022), low educational status (Cerda and Garcia, 2021), bad health condition (Erdogan et al., 2022) could have high level of fear of COVID-19.

Therefore, we assume that the characteristics factors for fear of COVID-19 include the following: gender, age, education status, job conditions, disease status and so on. These factors will serve as control factors that might affect the dependent variables in this research.

## Objective and hypotheses of current research

**Figure 1:**
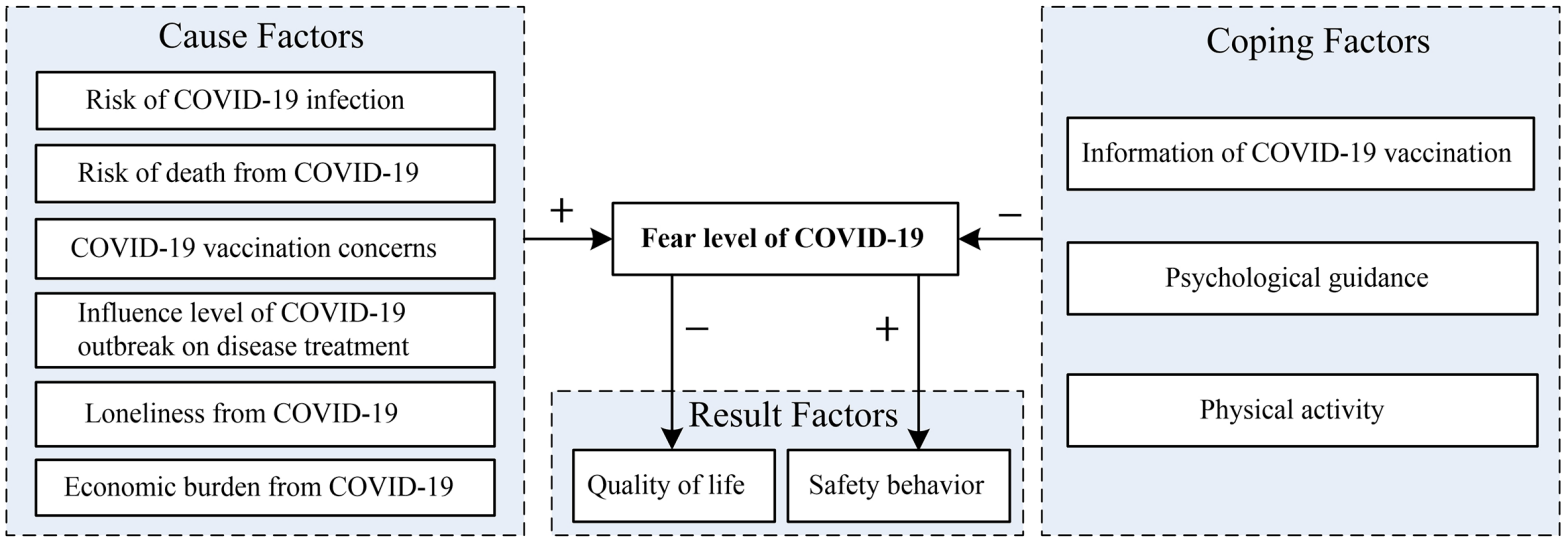
Theoretical model for predicting fear level of COVID-19.

The current research aims to identify the causes, results, and coping factors that predict an individual’s fear level of COVID-19 during the pandemic-related lockdown. This study focuses on a model (Figure 1) that investigates the relationship between cause factors (risk of COVID-19 infection, risk of death from COVID-19, COVID-19 vaccination concerns, influence level of COVID-19 outbreak on disease treatment, loneliness due to COVID-19, and economic burden from COVID-19), results factors (quality of life and safety behavior), coping factors (information regarding COVID-19 vaccination, psychological guidance, and physical activities), and COVID-19 fear level. The literature review indicates that the fear level can affect the quality of life and safe behavior of individuals, while the other factors affect fear level. Therefore, on the basis of the literature, the hypotheses are as follows.

*H1*: Risk of COVID-19 infection, risk of death from COVID-19, COVID-19 vaccination concerns, influence level of COVID-19 outbreak on disease treatment, loneliness due to COVID-19, and economic burden from COVID-19 are positively associated with COVID-19 fear level.

*H2*: Information regarding COVID-19 vaccination, psychological guidance, and physical activities are negatively associated with COVID-19 fear level.

*H3*: COVID-19 fear level is negatively associated with quality of life.

*H4*: COVID-19 fear level is positively associated with safety behavior.

## Methods

### Sample population

This cross-sectional study was conducted from December 2021 to March 2022 through the Wen Juan Xing platform, a famous online survey platform in China. A web-based survey was created for cancer inpatients in Henan Province, which has a higher prevalence of cancer in China. The participants received the questionnaire via WeChat (a Chinese social media and messaging application), and they responded using the “Quick Response code” on their mobile phones.

We used the following Cochrane formula to determine the minimum sample size for this study:


$$N=\frac{Z^{2}pq}{d^{2}}$$

where N is the sample size; p is the incidence of cancer among the population in Henan Province (data from the Henan cancer registration annual report in 2021); q is 1 – p ; Z is the standard normal deviation that is typically set at 1.96, corresponding to the 95% confidence interval (*CI*); and d is the degree of desired accuracy, which is set at 0.03 in this study. Therefore, the computed minimum sample size was 846. However, assuming that 10% of the questionnaire responses would be incomplete, the minimum sample size was set at 930 participants.

In this survey, our target population was recruited from two provincial hospitals because of the large number of cross-regional patients, such as city, county, township, or village. Patients with cancer were recruited through the chief nurse of each inpatient area in the sample hospitals. The inclusion criteria were as follows: age ≥ 18 years and living in Henan Province. All cancer patients admitted to the hospitals who met the inclusion criteria and agreed to participate in the survey were included. Patients who could not answer questions because of their mental state or presented severe clinical symptoms were excluded. A total of 1,067 cancer patients completed the survey (meeting the minimum sample requirements). The protocol for the current study was approved by the Life Science Ethics Review Committee of the Zhengzhou University (Approval number: 2021-01-12-05).

## Data measures

### COVID-19 fear level

COVID-19 fear level was measured using the Fear of COVID-19 Scale (FCV-19S) (Heyne et al., 2022). FCV-19S achieved good internal consistency in this study (Cronbach’s α=0.85 ). FCV-19S include seven items, such as “You are very afraid of COVID-19” and “The thought of COVID-19 makes you feel uncomfortable.” The participants indicated their level of agreement on a five-point Likert scale ranging from 1 (strongly agree) to 5 (strongly disagree). The total score was calculated by adding the score of each item. The average scores were then divided into the high, moderate, and low groups.

### Causal factors

The causal factors included the following: (1) risk of COVID-19 infection, (2) risk of death from COVID-19, (3) COVID-19 vaccination concerns, (4) influence level of COVID-19 outbreak on disease treatment, (5) loneliness due to COVID-19, and (6) economic burden from COVID-19. The participants indicated their perception level from “high,” “medium” and “low.”

### Coping factors

The coping factors included the following: (1) information regarding COVID-19 vaccination, (2) psychological guidance, and (3) physical activities. Among them, COVID-19 vaccination concerns were assessed using four items, and this variable exhibited good internal consistency in the current study (Cronbach’s α=0.70 ).

### Result factors

The result factors of fear of COVID-19 among cancer patients, the dependent variables were quality of life and safety behavior. The degree of quality of life among cancer patients was measured using EQ-5D, which included five items. This variable presented good internal consistency in this study (Cronbach’s α=0.85 ). The degree of safety behavior was measured using five items in accordance with COVID-19 prevention and control measures issued by the National Health Commission in China. It also showed good internal consistency in this study (Cronbach’s α=0.75 ). The participants indicated their level of agreement on a five-point Likert scale ranging from 1 (strongly agree) to 5 (strongly disagree). The total score was calculated by adding the score of each item. The average scores were divided into the high, moderate, and low groups.

### Control variables

The control variables included those that might affect the dependent variables on the basis of previous research, namely, individual characteristics (i.e., gender, age, education, and residential area) and disease status (i.e., type of cancer and status of cancer metastasis).

### Statistical analysis

All statistical analyses were performed using SPSS 26.0. All variables were presented as frequency distribution and percentage. The chi-square test was employed to investigate the corresponding associations of COVID-19 fear level, degree of quality of life, and safety behavior with the independent and control variables. Only variables with statistically significant differences were included in the cumulative logistic regression model. Variables with p<0.05 (two-tailed) were considered statistically significant.

## 1. Results

### 1.1. Sample characteristics

The characteristics of the study population are provided in Table 1. The proportion of males (52.1%) was higher than that of females (47.9%). The majority of the participants were older than 50 years (64.5%) and had an education status above junior high school level (76.0%). The proportion of participants who came from rural areas was higher (64.0%) than that from urban areas (36.0%). The majority of the participants were married (90.8%). Nearly half of participants had jobs (47.2%). In this survey, nearly half (48.0%) of the inpatients were diagnosed with cancer within 1 year. The major cancer types were digestive system cancer (42.5%), followed by respiratory system cancer (15.9%) and mammary gland system cancer (11.7%). The majority of the participants did not experience cancer metastases (87.9%), and 61.7% were hospitalized for more than 7 days.

**Table 1:** Distribution of Individual characteristics, causal factors, result factors, coping factors and COVID-19 fear level in this study’s sample (*N* = 1,067).

Variables	All participants (%)	Fear level of COVID-19				
		High (%)	Middle (%)	Low (%)	*χ^2^*	*p-*Value
Total participants	*n*=1,067 (100)	*n*=207 (19.4)	*n*=714 (66.9)	*n*=146 (13.7)		
*Demographic characteristics*						
Gender					7.147	0.028
Male	556 (52.1)	117 (21.0)	352 (63.3)	87 (15.6)		
Female	511 (47.9)	90 (17.6)	362 (70.8)	59 (11.5)		
Age					5.654	0.686
18–29	55 (5.2)	12 (21.8)	22 (60.0)	10 (18.2)		
30–39	130 (12.2)	25 (19.2)	93 (71.5)	12 (9.2)		
40–49	194 (18.2)	36 (18.6)	128 (66.0)	30 (15.5)		
50–59	342 (32.1)	65 (19.0)	235 (68.7)	42 (12.3)		
≥ 60	346 (32.4)	69 (20.0)	224 (64.9)	52 (15.1)		
Education status					15.827	0.015
Primary school and below	257 (24.1)	40 (15.6)	177 (68.9)	40 (15.6)		
Junior high school	371 (34.8)	63 (17.0)	248 (66.8)	60 (16.2)		
Senior high school	272 (25.5)	58 (21.3)	183 (67.3)	31 (11.4)		
College school and above	167 (15.7)	46 (27.5)	106 (63.5)	15 (9.0)		
Residential area					0.223	0.895
City	384 (36.0)	75 (19.5)	259 (67.4)	50 (13.0)		
Rural	683 (64.0)	132 (19.3)	455 (66.6)	96 (14.1)		
Marital status					1.962	0.375
Married	969 (90.8)	193 (19.9)	643 (66.4)	133 (13.7)		
Others	98 (9.2)	14 (14.3)	71 (72.4)	13 (13.3)		
Job conditions					8.341	0.015
Job	504 (47.2)	115 (22.8)	329 (65.3)	60 (11.9)		
Jobless	563 (52.8)	92 (16.3)	385 (68.4)	86 (15.3)		
*Disease status*						
Duration of cancer diagnosis					7.796	0.253
Less than one year	512 (48.0)	85 (16.6)	355 (69.3)	72 (14.1)		
One year	303 (28.4)	69 (22.8)	196 (64.7)	38 (12.5)		
Two years	91 (8.5)	18 (19.8)	56 (61.5)	17 (18.7)		
Three years and above	161 (15.1)	35 (21.7)	107 (66.5)	19 (11.8)		
Types of cancer					13.025	0.525
Digestive system	453 (42.5)	98 (21.6)	296 (65.3)	59 (13.0)		
Respiratory system	170 (15.9)	26 (15.3)	114 (67.1)	30 (17.6)		
Mammary gland system	125 (11.7)	18 (14.4)	93 (74.4)	14 (11.2)		
Genitourinary system	39 (3.7)	9 (23.1)	24 (61.5)	6 (15.4)		
Endocrine system	60 (5.6)	11 (18.3)	40 (66.7)	9 (15.0)		
Nervous system	102 (9.6)	23 (22.5)	68 (66.7)	11 (10.8)		
Circulatory system	97 (9.1)	16 (16.5)	65 (67.0)	16 (16.5)		
Other	21 (2.0)	6 (28.6)	14 (66.7)	1 (4.8)		
Status of cancer metastasis					2.413	0.299
No	938 (87.9)	181 (19.3)	634 (67.6)	123 (13.1)		
Yes	129 (12.1)	26 (20.2)	80 (62.0)	23 (17.8)		
Days of hospitalization					10.416	0.034
≤ 3 days	166 (15.6)	31 (18.7)	117 (70.5)	18 (10.8)		
4–6 days	244 (22.9)	39 (16.0)	180 (73.8)	25 (10.2)		
≥7 days	657 (61.6)	137 (20.9)	417 (63.5)	103 (15.7)		
*Causal factors of fear of COVID-19*						
Risk of COVID-19 infection					41.382	<0.001
High risk	135 (12.7)	45 (33.3)	80 (59.3)	10 (7.4)		
Medium risk	149 (14.0)	20 (13.4)	107 (71.8)	22 (14.8)		
Low risk	543 (50.9)	116 (21.4)	362 (66.7)	65 (12.0)		
Do not know	240 (22.5)	26 (10.8)	165 (68.8)	49 (20.4)		
Risk of death from COVID-19					51.474	<0.001
High risk	551 (51.6)	145 (26.3)	346 (62.8)	60 (10.9)		
Medium risk	203 (19.0)	31 (15.3)	146 (71.9)	26 (12.8)		
Low risk	91 (8.5)	18 (19.8)	59 (64.8)	14 (15.4)		
Do not know	222 (20.8)	13 (5.9)	163 (73.4)	46 (20.7)		
Worry level of COVID-19 vaccination					38.751	<0.001
High	215 (20.1)	61 (28.4)	132 (61.4)	22 (10.2)		
Medium	684 (64.1)	121 (17.7)	483 (70.6)	80 (11.7)		
Low	168 (15.7)	25 (14.9)	99 (58.9)	44 (26.2)		
Influence level of COVID-19 outbreak on disease treatment					35.232	<0.001
High	692 (64.9)	161 (23.3)	459 (66.3)	72 (10.4)		
Medium	254 (23.8)	37 (14.6)	172 (67.7)	45 (17.7)		
Low	121 (11.3)	9 (7.4)	83 (68.6)	29 (24.0)		
Loneliness from COVID-19					29.306	<0.001
Serious	83 (7.8)	33 (39.8)	40 (48.2)	10 (12.0)		
General	384 (36.0)	64 (16.7)	255 (66.4)	65 (16.9)		
Not serious	600 (56.2)	110 (18.3)	419 (69.8)	71 (11.8)		
Economic burden from COVID-19					25.959	<0.001
Serious	506 (47.4)	120 (23.7)	321 (63.4)	65 (12.8)		
General	346 (32.4)	55 (15.9)	256 (74.0)	35 (10.1)		
Not serious	215 (20.1)	32 (14.9)	137 (63.7)	46 (21.4)		
*Result factors of fear of COVID-19*						
Quality of life					13.362	0.010
Better	309 (29.0)	42 (13.6)	222 (71.8)	45 (14.6)		
General	588 (55.1)	119 (20.2)	390 (66.3)	79 (13.4)		
Worse	170 (15.9)	46 (27.1)	102 (60.0)	22 (12.9)		
Safety behavior					64.029	<0.001
Better	216 (20.2)	75 (34.7)	120 (55.6)	21 (9.7)		
General	714 (66.9)	123 (17.2)	502 (70.3)	89 (12.5)		
Worse	137 (12.8)	9 (6.6)	92 (67.2)	36 (26.3)		
*Coping factors of fear of COVID-19*						
Information of COVID-19 vaccination					291.618	<0.001
Unobtainable	585 (54.8)	187 (32.0)	342 (58.6)	55 (9.4)		
Not sure	331 (31.0)	12 (3.6)	299 (90.3)	20 (6.0)		
Obtainable	151 (14.2)	8 (5.3)	72 (47.7)	71 (47.0)		
Psychological guidance					49.223	<0.001
Unobtainable	842 (78.9)	189 (22.4)	544 (64.6)	109 (12.9)		
Not sure	112 (10.5)	1 (0.9)	102 (91.1)	9 (8.0)		
Obtainable	113 (10.6)	17 (15.0)	68 (60.2)	28 (24.8)		
Physical Activity					11.375	0.023
Less or never	421 (39.5)	94 (22.3)	286 (67.9)	41 (9.7)		
General	331 (31.0)	60 (18.1)	219 (66.2)	52 (15.7)		
Always	315 (29.5)	53 (16.8)	209 (66.3)	53 (16.8)		

### Prevalence of COVID-19 fear level

**Figure 2:**
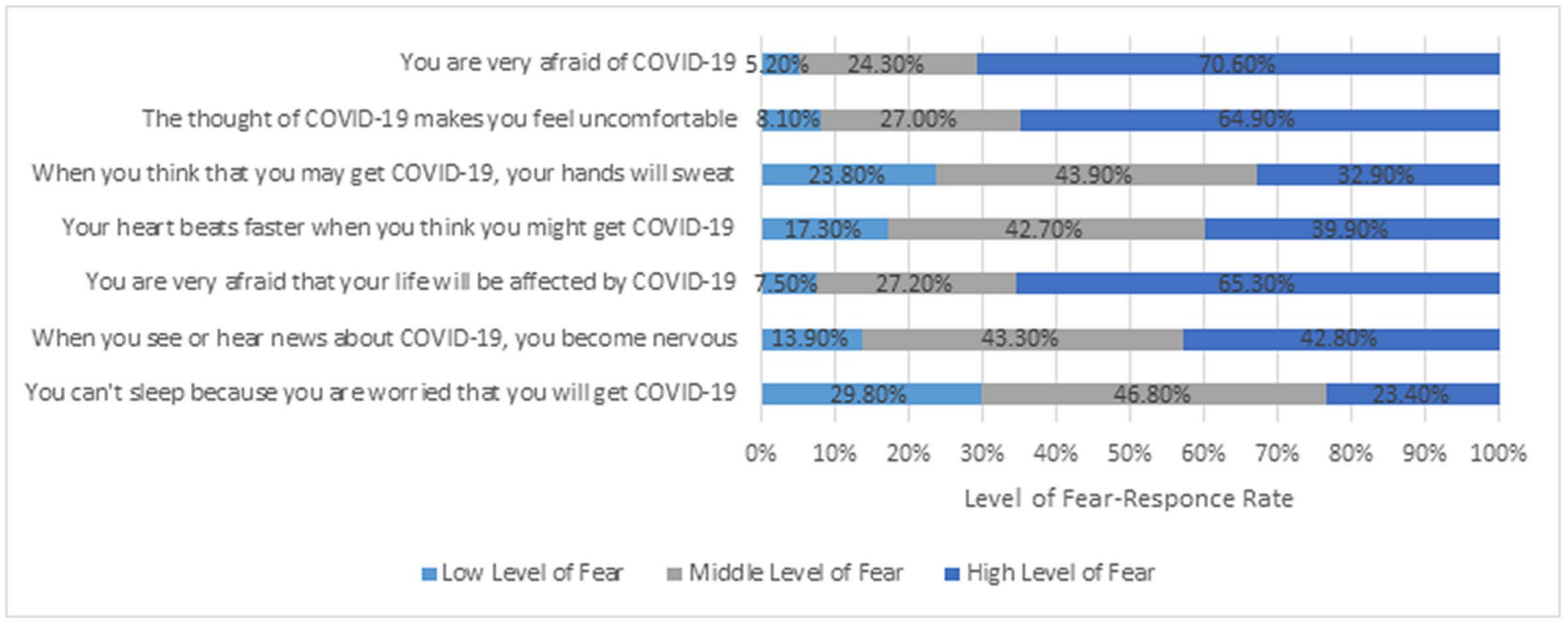
Distribution of responses on fear of COVID-19 items (N = 1,067).

Among the 1,067 cancer patients, 207 expressed a high level of COVID-19 fear (19.4%), while 714 (66.9%) and 146 (13.7%) expressed moderate and low levels of COVID-19 fear, respectively. In terms of specific items (Figure 2), the fear of COVID-19 of cancer patients was mostly manifested through psychological reactions, such as feeling uncomfortable (64.9%), and less through physiological reactions caused by fear, such as sweating hands (32.9%), faster heartbeat (39.9%), or difficulty sleeping (23.4%).

A significant difference was found in COVID-19 fear level in relation to various control variables. A higher fear level of COVID-19 was observed among participants who were female (*χ^2^* = 7.147, *p* = 0.028), had a high level of education (*χ^2^* = 15.827, *p* = 0.015), had a job (*χ^2^* = 8.341, *p* = 0.015), and had a long period of hospitalization (*χ^2^* = 10.416, *p* = 0.034). Meanwhile, the research showed that causal, result, and coping factors were associated with COVID-19 fear level. A higher fear level of COVID-19 was observed among participants who had higher risk of COVID-19 infection (*χ^2^* = 41.382, *p*<0.001), higher risk of death due to COVID-19 (*χ^2^* = 51.474, *p*<0.001), more concerns regarding COVID-19 vaccination (*χ^2^* = 38.751, *p*<0.001), higher influence on their disease treatment (*χ^2^* = 35.232, *p*<0.001), severe loneliness due to COVID-19 (*χ^2^* = 29.306, *p*<0.001), serious economic burden from COVID-19 (*χ^2^* = 25.959, *p*<0.001), worse quality of life (*χ^2^* = 13.362, *p* = 0.010), better safety behavior (*χ^2^* = 64.029, *p*<0.001), could not obtain information regarding COVID-19 vaccination (*χ^2^* = 291.618, *p*<0.001), no psychological guidance (*χ^2^* = 49.223, *p*<0.001), and less or no daily physical activities (*χ^2^* = 11.375, *p* = 0.023). The detailed results are provided in Table 1.

### Causes of fear of COVID-19

In this study, half of the participants believed that they had a low risk of COVID-19 infection (50.9%). However, more than half of the cancer patients believed that they had a high risk of dying once they got infected with COVID-19 (51.6%). The majority of the patients had moderate (64.1%) or high (20.1%) level of fear toward COVID-19 vaccination. The majority of the cancer patients believed that the COVID-19 pandemic exerted a high level of influence on their disease treatment (64.9%). Simultaneously, half of the cancer patients expressed a low level of loneliness (56.2%), but nearly half of the participants experienced serious economic burden because of the COVID-19 pandemic (47.4%).

After discovering that gender, education status, job conditions, and days of hospitalization were significantly associated with COVID-19 fear level, these factors were controlled using cumulative logistic regression analysis to examine the causal factors of COVID-19 fear level. The participants who believed that they had a high risk of COVID-19 infection had a higher fear level than patients who unclearly expressed their risk of COVID-19 infection (*β* = −0.551, 95% *CI* = −1.065 to −0.038, *p* = 0.035). Compared with those who did not know their risk of death due to COVID-19, respondents who had a high (*β* = −0.927, 95% *CI* = −1.348 to −0.507, *p*<0.001), moderate (*β* = −0.643, 95% *CI* = −1.125 to −0.161, *p* = 0.009), and low (*β* = −0.711, 95% *CI* = −1.302 to −0.120, *p* = 0.018) risk of death when infected with COVID-19 had a higher level of fear of COVID-19. Respondents with high (*β* = −1.260, 95% *CI* = −1.709 to −0.810, *p*<0.001) and moderate (*β* = −0.791, 95% *CI* = −1.164 to −0.418, *p*<0.001) levels of concern toward COVID-19 vaccination had a higher fear level of COVID-19 than those with a low level of concern. During the COVID-19 pandemic, patients had high (*β* = −0.941, 95% *CI* = −1.371 to −0.511, *p*<0.001) and moderate (*β* = −0.540, 95% *CI* = −1.013 to −0.068, *p* = 0.025) levels of disturbance of their disease treatment apparently had a higher level of COVID-19 fear than those with a low level of disturbance of their disease treatment. Patients who experienced serious loneliness because of COVID-19 had a higher fear level of COVID-19 than those who experienced a low level of loneliness (*β* = −0.816, 95% *CI* = −1.311 to −0.321, *p* = 0.001). Compared with the respondents whose economic status was less affected by COVID-19, participants who had serious (*β* = −1.094, 95% *CI* = −1.467 to −0.722, *P*<0.001) and general (*β* = −0.693, 95% *CI* = −1.069 to −0.317, *p*<0.001) economic burden from COVID-19 were more afraid of the COVID-19 pandemic. The detailed results are provided in Table 2.

**Table 2:** Outcome of a cumulative logistic regression model examining causal factors of COVID-19 fear level.

Variables	*B*	*p*-Value	*OR*	95%CI	
				Lower	Upper
Control variables					
*Gender*					
Male	0.085	0.526	1.088	−0.177	0.347
Female	Ref.				
*Education status*					
Primary school and below	0.791	0.001	2.206	0.338	1.244
Junior high school	0.649	0.003	1.914	0.227	1.072
Senior high school	0.272	0.206	1.313	−0.150	0.694
College school and above	Ref.				
*Job conditions*					
Job	−0.388	0.004	0.678	−0.653	−0.123
Jobless	Ref.				
*Days of hospitalization*					
≤3 days	−0.069	0.713	0.933	−0.435	0.297
4–6 days	−0.018	0.909	0.982	−0.335	0.299
≥7 days	Ref.				
Independent variables					
*Risk of COVID-19 infection*					
High risk	−0.551	0.035	0.576	−1.065	−0.038
Medium risk	0.208	0.430	1.231	−0.309	0.724
Low risk	−0.287	0.154	0.751	−0.683	0.108
Do not know	Ref.				
*Risk of death from COVID-19*					
High risk	−0.927	<0.001	0.396	−1.348	−0.507
Medium risk	−0.643	0.009	0.526	−1.125	−0.161
Low risk	−0.711	0.018	0.491	−1.302	−0.120
Do not know	Ref.				
*Worry level of COVID-19 vaccination*					
High	−1.260	<0.001	0.284	−1.709	−0.810
Medium	−0.791	<0.001	0.453	−1.164	−0.418
Low	Ref.				
*Influence level of COVID-19 outbreak on disease treatment*					
High	−0.941	<0.001	0.390	−1.371	−0.511
Medium	−0.540	0.025	0.583	−1.013	−0.068
Low	Ref.				
*Loneliness from COVID-19*					
Serious	−0.816	0.001	0.442	−1.311	−0.321
General	0.029	0.845	1.029	−0.258	0.315
Not serious	Ref.				
*Economic burden from COVID-19*					
Serious	−1.094	<0.001	0.335	−1.467	−0.722
General	−0.693	<0.001	0.500	−1.069	−0.317
Not serious	Ref.				

### Results of fear of COVID-19

Among the 1,067 cancer patients, half of the participants had general (50.9%) or better (14.0%) quality of life, while 15.9% respondents had poor quality of life. During the COVID-19 pandemic, the majority of the participants tended to practice safety behavior (87.1%), while 12.8% of the patients had negative protective behavior. A significant difference was found between quality of life and safety behavior on various control variables. Better quality of life was observed among participants who had a shorter length of cancer diagnosis (*χ^2^* = 15.735, *p* = 0.015) and shorter days of hospitalization (*χ^2^* = 17.594, *p* = 0.001). Better safety behavior was observed among participants who were female (*χ^2^* = 6.016, *p* = 0.049) and had higher educational background (*χ^2^* = 13.624, *p* = 0.034). The detailed results are presented in Table 3.

**Table 3:** Distribution of Individual characteristics, disease status and coping factors of COVID-19 fear level in this study’s sample (*N* = 1,067).

Variables	Quality of life			*χ^2^* (*p-*Value)	Safety behavior			*χ^2^* (*p-*Value)
	Better (%)	General (%)	Worse (%)		Better (%)	General (%)	Worse (%)	
Demographic characteristics								
Gender								
Male	167 (30.0)	306 (55.0)	83 (14.9)	1.201 (0.549)	102 (18.3)	371 (66.7)	83 (14.9)	6.016 (0.049)
Female	142 (27.8)	282 (55.2)	87 (17.0)		114 (22.3)	343 (67.1)	54 (10.6)	
Age								
18–29	19 (34.5)	28 (50.9)	8 (14.5)	14.872 (0.062)	13 (23.6)	35 (63.6)	7 (12.7)	3.357 (0.910)
30–39	49 (37.7)	69 (53.1)	12 (9.2)		32 (24.6)	84 (64.6)	14 (10.8)	
40–49	57 (29.4)	112 (57.7)	25 (12.9)		34 (17.5)	134 (69.1)	26 (13.4)	
50–59	99 (28.9)	186 (54.4)	57 (16.7)		70 (20.5)	226 (66.1)	46 (13.5)	
≥ 60	85 (24.6)	192 (55.7)	68 (19.7)		67 (19.4)	234 (67.8)	44 (12.8)	
Education status								
Primary school and below	73 (28.4)	134 (52.1)	50 (19.5)	7.309 (0.293)	44 (17.1)	164 (63.8)	49 (19.1)	13.624 (0.034)
Junior high school	102 (27.5)	209 (56.3)	60 (16.2)		82 (22.1)	246 (66.3)	43 (11.6)	
Senior high school	79 (29.0)	150 (55.1)	43 (15.8)		54 (19.9)	188 (69.1)	30 (11.0)	
College school and above	55 (32.9)	95 (56.9)	17 (10.2)		36 (21.6)	116 (69.5)	15 (9.0)	
Residential area								
City	114 (29.7)	217 (56.5)	53 (13.8)	2.033 (0.362)	83 (21.6)	249 (64.8)	52 (13.5)	1.172 (0.556)
Rural	195 (28.6)	371 (54.3)	117 (17.1)		133 (19.5)	465 (68.1)	85 (12.4)	
Marital status								
Married	282 (29.1)	531 (54.8)	156 (16.1)	0.440 (0.802)	198 (20.4)	645 (66.6)	126 (13.0)	0.604 (0.740)
Others	27 (27.6)	57 (58.2)	14 (14.3)		18 (18.4)	69 (70.4)	11 (11.2)	
Job conditions								
Job	145 (28.8)	280 (55.6)	79 (15.7)	0.087 (0.958)	95 (18.8)	339 (67.3)	70 (13.9)	1.753 (0.416)
Jobless	164 (29.1)	308 (54.7)	91 (16.2)		121 (21.5)	375 (66.6)	67 (11.9)	
Disease status								
Duration of cancer diagnosis								
Less than one year	161 (31.4)	285 (55.7)	66 (12.9)	15.735 (0.015)	102 (19.9)	343 (67.0)	67 (13.1)	1.190 (0.977)
One year	93 (30.7)	162 (53.5)	48 (15.8)		63 (20.8)	198 (65.3)	42 (13.9)	
Two years	20 (22.0)	49 (53.8)	22 (24.2)		19 (20.9)	62 (68.1)	10 (11.0)	
Three years and above	35 (21.7)	92 (57.1)	34 (21.1)		32 (19.9)	111 (68.9)	18 (11.2)	
Types of cancer								
Digestive system	111 (24.5)	261 (57.6)	81 (17.9)	38.036 (0.001)	99 (21.9)	310 (68.4)	44 (9.7)	26.693 (0.021)
Respiratory system	51 (30.0)	94 (55.3)	25 (14.7)		24 (14.1)	119 (70.0)	27 (15.9)	
Mammary gland system	33 (26.4)	76 (60.8)	16 (12.8)		23 (18.4)	84 (67.2)	18 (14.4)	
Genitourinary system	7 (17.9)	27 (69.2)	5 (12.8)		9 (23.1)	20 (51.3)	10 (25.6)	
Endocrine system	19 (31.7)	35 (58.3)	6 (10.0)		15 (25.0)	33 (55.0)	12 (20.0)	
Nervous system	50 (31.7)	40 (39.2)	12 (11.8)		27 (26.5)	62 (60.8)	13 (12.7)	
Circulatory system	33 (34.0)	42 (43.3)	22 (22.7)		18 (18.6)	69 (71.1)	10 (10.3)	
Other	5 (23.8)	13 (61.9)	3 (14.3)		1 (4.8)	17 (81.0)	3 (14.3)	
Status of cancer metastasis								
No	280 (29.9)	509 (54.3)	149 (15.9)	3.140 (0.208)	183 (19.5)	635 (67.7)	120 (12.8)	2.785 (0.248)
Yes	29 (22.5)	79 (61.2)	21 (16.3)		33 (25.6)	79 (61.2)	17 (13.2)	
Days of hospitalization								
≤ 3 days	58 (34.9)	91 (54.8)	17 (10.2)	17.594 (0.001)	27 (16.3)	121 (72.9)	18 (10.8)	3.350 (0.501)
4–6 days	67 (27.5)	151 (61.9)	26 (10.7)		50 (20.5)	163 (66.8)	31 (12.7)	
≥7 days	184 (28.0)	346 (52.7)	127 (19.3)		139 (21.2)	430 (65.4)	88 (13.4)	

After discovering that the duration of cancer diagnosis, types of cancer, and days of hospitalization were significantly associated with quality of life, while gender, education status, and types of cancer were significantly associated with safety behavior, these factors were controlled using cumulative logistic regression analysis to examine the result factors on COVID-19 fear level. The results revealed that compared with patients with low fear level of COVID-19, respondents with a high level of COVID-19 fear apparently had worse quality of life (*β* = 0.548, 95% *CI* = 0.132 to 0.965, *p* = 0.010). Participants with high (*β* = −1.560, 95% *CI* = −2.020 to −1.100, *P*<0.001) or moderate (*β* = −0.514, 95% *CI* = −0.897 to −0.131, *p* = 0.009) COVID-19 fear level could demonstrate better safety behavior than those with low fear level. The detailed results are provided in Tables 4, 5.

**Table 4:** Outcome of a cumulative logistic regression model examining COVID-19 fear level of quality of life.

Variables	*B*	*p*-Value	*OR*	95%CI	
				Lower	Upper
Control variables					
Duration of cancer diagnosis					
Less than one year	−0.484	0.007	0.616	−0.839	−0.130
One year	−0.399	0.038	0.671	−0.776	−0.022
Two years	0.077	0.763	1.080	−0.425	−0.580
Three years and above	Ref.				
Types of cancer					
Digestive system	0.031	0.944	1.031	−0.823	0.885
Respiratory system	−0.182	0.688	0.834	−1.068	0.704
Mammary gland system	−0.038	0.933	0.963	−0.940	0.863
Genitourinary system	−0.004	0.994	0.996	−1.041	1.034
Endocrine system	−0.386	0.436	0.680	−1.356	0.585
Nervous system	−0.845	0.071	0.430	−1.762	0.072
Circulatory system	−0.164	0.727	0.849	−1.087	0.759
Other	Ref.				
Days of hospitalization					
≤ 3 days	−0.444	0.010	0.641	−0.781	−0.108
4–6 days	−0.241	0.104	0.786	−0.532	0.050
≥7 days	Ref.				
Independent variables					
Fear level of COVID-19					
High	0.548	0.010	1.730	0.132	0.965
Medium	0.022	0.902	1.022	−0.327	0.370
Low	Ref.				

**Table 5:** Outcome of a cumulative logistic regression model examining COVID-19 fear level of safety behavior.

Variables	*B*	*p*-Value	*OR*	95%CI	
				Lower	Upper
Control variables					
Gender					
Male	0.488	0.001	1.629	0.210	0.766
Female	Ref.				
Education status					
Primary school and below	0.427	0.047	1.533	0.005	0.849
Junior high school	−0.078	0.695	0.924	−0.468	0.312
Senior high school	0.029	0.890	1.029	−0.379	0.437
College school and above	Ref.				
Types of cancer					
Digestive system	−1.025	0.030	0.359	−1.950	−0.100
Respiratory system	−0.584	0.232	0.558	−1.542	0.374
Mammary gland system	−0.506	0.313	0.603	−1.490	0.478
Genitourinary system	−0.301	0.598	0.740	−1.421	0.819
Endocrine system	−0.782	0.144	0.457	−1.833	0.268
Nervous system	−0.963	0.057	0.382	−1.955	0.028
Circulatory system	−0.856	0.093	0.425	−1.853	0.141
Other	Ref.				
Independent variables					
Fear level of COVID-19					
High	−1.560	<0.001	0.210	−2.020	−1.100
Medium	−0.514	0.009	0.598	−0.897	−0.131
Low	Ref.				

### Coping with fear of COVID-19

In terms of coping measures, approximately half (54.8%) of the participants expressed that personalized information regarding COVID-19 vaccine and psychological guidance (78.9%) could not be obtained. Only 29.5% of the participants had physical activities.

After discovering that gender, education status, job conditions, and days of hospitalization were significantly associated with COVID-19 fear level, these factors were controlled for using cumulative logistic regression analysis to examine the causal factors on COVID-19 fear level.

Cancer patients who could not obtain personalized information regarding COVID-19 vaccination (*β* = −2.983, 95% *CI* = −3.424 to −2.542, *p*<0.001) and psychological guidance (*β* = −1.461, 95% *CI* = −1.931 to −0.991, *p*<0.001) had a higher level of fear than the respondents who could obtain them. Compared with participants who had better physical activities, the respondents who exercised less or never exercised had a high level of COVID-19 fear (*β* = −0.431, 95% *CI* = −0.753 to −0.109, *p* = 0.009). The detailed results are provided in Table 6.

**Table 6:** Outcome of a cumulative logistic regression model examining coping factors of COVID-19 fear level.

Variables	*B*	*p*-Value	*OR*	95%CI	
				Lower	Upper
Control variables					
Gender					
Male	−0.006	0.966	0.994	−0.269	0.257
Female	Ref.				
Education status					
Primary school and below	0.353	0.105	1.423	−0.074	0.779
Junior high school	0.447	0.028	1.564	0.049	0.884
Senior high school	0.206	0.328	1.229	−0.207	0.619
College school and above	Ref.				
Job conditions					
Job	−0.287	0.034	0.751	−0.552	−0.021
Jobless	Ref.				
Days of hospitalization					
≤3 days	0.024	0.897	1.024	−0.342	0.391
4–6 days	−0.013	0.938	0.987	−0.330	0.305
≥7 days	Ref.				
Independent variables					
Information of COVID-19 vaccination					
Unobtainable	−2.983	<0.001	0.051	−3.424	−2.542
Not sure	−1.711	<0.001	0.181	−2.137	−1.286
Obtainable	Ref.				
Psychological guidance					
Unobtainable	−1.461	<0.001	0.232	−1.931	−0.991
Not sure	−0.869	0.004	0.419	−1.468	−0.270
Obtainable	Ref.				
Physical Activity					
Less or never	−0.431	0.009	0.650	−0.753	−0.109
General	−0.137	0.427	0.872	−0.475	0.201
Always	Ref.				

## Discussion

This study indicates that cancer patients reported a moderate level of fear of COVID-19 in Central China. During the lockdown, the majority of cancer patients were psychologically afraid and had no corresponding physiological reaction. However, about 30% of the respondents had already presented physiological responses, such as sweaty hands, fast heartbeat, and difficulty sleeping, because of fear of COVID-19 infection. This phenomenon is threatening the disease rehabilitation of cancer patients. Furthermore, females experienced higher levels of fear of COVID-19 than males. This finding is parallel to a meta-analysis of gender and fear of COVID-19 (Metin et al., 2022). Cancer patients with a high level of education tend to have higher fear level of COVID-19 than others. This result is contrary to those of previous studies on the general population (Cerda and Garcia, 2021). Similarly, the result showed that employed cancer patients had higher COVID-19 fear level than those who were unemployed; the difference is from Inna Levy and Keren Cohen-Louck (Levy and Cohen-Louck, 2021). This result could be attributed to employed patients being more afraid of unemployment caused by COVID-19. Meanwhile, this study revealed that cancer patients with a long period of hospitalization had a high level of COVID-19 fear. This finding suggests that the attending physician should pay attention to the mental health of such patients in addition to disease treatment.

This study constructed a model that included six cause factors, two result factors, three coping factors, and fear level of COVID-19. The data supported our hypotheses. This study’s major findings indicate that six cause factors (risk of COVID-19 infection, risk of death from COVID-19, COVID-19 vaccination concerns, influence level of COVID-19 outbreak on disease treatment, loneliness due to COVID-19, and economic burden from COVID-19) are positively associated with COVID-19 fear level. Meanwhile, three coping factors (information regarding COVID-19 vaccination, psychological guidance, and physical activities) are negatively associated with COVID-19 fear level. In terms of result factors, COVID-19 fear level is negatively associated with quality of life and positively associated with safety behavior.

Our findings indicated that individuals had a high fear level of COVID-19 when they thought they had a high risk of COVID-19 infection, high risk of death from COVID-19, high concern level toward COVID-19 vaccination, and high influence level of COVID-19 outbreak on disease treatment. These factors are primarily related to the health status of the participants. For cancer patients, the COVID-19 pandemic exacerbates their concerns about their own health problems, particularly when the effectiveness of COVID-19 vaccination may be insufficient or have COVID-19 vaccination contraindications. In addition, 64.9% of cancer patients experienced a high level of treatment delays due to COVID-19 in the present research. A series of effort had been exerted to stop the spread of COVID-19 worldwide, although these policies could disturb normal medical behavior. Hence, this finding is in line with a survey in the Netherlands whereby chemotherapy, immunotherapy, or radiotherapy for many cancer patients was postponed or cancelled (Guven et al., 2020a; van der Veldt et al., 2021; Barik et al., 2022). However, patients’ fear of COVID-19 grew as a result of the delay in treatment. Providing normal health care services during the COVID-19 pandemic is a practical problem that must be solved urgently. Furthermore, cancer patients who suffer from severe loneliness and economic burden from COVID-19 had a high level of fear of COVID-19. This result is consistent with a previous survey of the general population (Nurnberger et al., 2022). In China, most hospitals require that inpatients not be allowed to visit during the COVID-19 pandemic, increasing the loneliness of cancer patients. This result further support the previous content that cancer patients with a long period of hospitalization had a high level of COVID-19 fear, which could be associated with being lonely. Meanwhile, the increasing economic burden caused by COVID-19 seems to have become a concern of people worldwide (Argabright et al., 2022). This issue could be more serious for cancer patients because they need to expend on disease treatment.

Our findings indicated that individuals with a high fear level of COVID-19 could have a worse quality of life and adopt active safety behavior. As defined by the World Health Organization, quality of life is based on people’s understanding of different aspects of their life. It is related to the value system of the country where they are living and their goals, expectations, standards, and priorities. During the COVID-19 pandemic, individuals of mental quality of life is directly affected by fear of COVID-19 that develop negative emotions and psychological problems. Then, other aspects could be affected, such as physical pain or decreased immunity. Hence previous research has the same results as the current study (Naghizadeh and Mirghafourvand, 2021; Demirbas and Kutlu, 2022). The decline in quality of life is one of the most serious consequences of the COVID-19 pandemic in addition to health status. Although fear of COVID-19 has exerts a considerable side effect on individuals, it still has some positive effects. This study demonstrated that fear COVID-19 will help individuals actively improve their health awareness and practice more effective safety behavior. This result is consistent with many theories, such as emotion–motivation model (Hein et al., 2022) or protection–motivation model (Bhati et al., 2021). For cancer patients, active safety behavior is an effective approach to protecting themselves from COVID-19 infection, particularly those with diseases that require delayed vaccination as per protocol. However, we must give attention to the non-recommended safety behavior due to excessive fear of COVID-19 (Kohler et al., 2021), such as excessive hoarding of food, which requires the joint effort of the whole society.

Furthermore, our research demonstrated the effectiveness of three mainstream coping measures in dealing with fear of COVID-19. First, providing professional COVID-19 vaccination advice to cancer patients is an effective way to reduce their fear. Although a large number of scientists worldwide believe that the safety of COVID-19 vaccines can be guaranteed (Gundavda and Gundavda, 2021), some cancer patients remain skeptical about the safety and effectiveness of COVID-19 vaccination, because the existing vaccination recommendations for cancer patients are an extension of the findings of the clinical trials based on the general population. Concurrently, COVID-19 vaccination suggestions vary for cancer patients with different types, which may lead to confusion among patients. As mentioned earlier, worry and confusion about COVID-19 vaccination will increase cancer patients’ fear level of COVID-19. Therefore, providing professional COVID-19 vaccination advice to cancer patients is a necessary measure for improving vaccination rate and reducing fear level of COVID-19. However, our research showed that only 14.2% of cancer patients have received professional advice on COVID-19 vaccination. Second, psychological counseling is also an internationally recognized effective measure to help individual reduce their fear level of COVID-19 (Loscalzo, 2022). Our research also proves this result. However, very few people receive psychological counseling at present. This research showed that 86.3% of respondents have medium and high levels of COVID-19 fear, while only 10.6% patients have used psychological guidance. Previous studies have also shown that cancer patients do not need psychological counseling, although they suffer from depression and anxiety (Mo et al., 2022). These results indicate that cancer patients do not recognize the importance of psychological counseling. Therefore, the health system should focus on how to improve the rate of providing personalized vaccine counseling and psychological guidance during the COVID-19 pandemic, such as undertaking this responsibility by their attending physicians. Finally, our finding indicated that physical activities also play a positive role on reducing the fear level of COVID-19. Previous studies have suggested that physical exercise as therapy can counteract or at least mitigate the mental and physical consequences of COVID-19-induced restrictive measures (Jimenez-Pavon et al., 2020). Therefore, some scholars have proposed that physical activities should be incorporated as part of cognitive behavior therapy during the COVID-19 pandemic (Ho et al., 2020).

## Limitations

This study has several shortcomings. First, the study only included participants who were under cancer treatment during the execution of the study. Meanwhile, data were collected via online questionnaires by utilizing the convenience sampling approach instead of probability sampling, limiting the representativeness of the whole population with cancer. Second, our research focused on analyzing six cause factors, two result factors, and three coping factors of COVID-19 fear level based on previous research and reality. However, factors related to the fear level of COVID-19 are complex and require further research in the future. Third, this study is based on self-reports; therefore, the results represent participant’s subjective assessment of some variables, such as influence level of COVID-19 outbreak on disease treatment. Therefore, future studies should consider examining our model by using objective data.

## Conclusion

The contributions of this study are theoretical and practical. It contributes to the body of literature by presenting a model for predicting the causes, results, and coping factors of COVID-19 fear level. In accordance with our model, individuals with a high risk of COVID-19 infection, high risk of death from COVID-19, high level of concern toward COVID-19 vaccination, high influence level of COVID-19 pandemic on disease treatment, severe loneliness, and economic burden from COVID-19 can lead to a high level of fear of COVID-19. Cancer patients with a high level of COVID-19 fear will have worse quality of life and active safety behavior. Furthermore, cancer patients with fear of COVID-19 can be reduced by obtaining information about COVID-19 vaccination, psychological guidance, and physical activities. Therefore, the authors suggest that governments should improve access to personalized vaccine counseling and psychological guidance during the COVID-19 pandemic by undertaking the responsibility of patients’ attending physicians and increasing publicity. Moreover, physical activities should be part of the treatment program to help cancer patients better recover their physical and mental health.

## Data availability statement

The original contributions presented in the study are included in the article/Supplementary material, further inquiries can be directed to the corresponding author.

## Ethics statement

The studies involving human participants were reviewed and approved by the Life Science Ethics Review Committee of the Zhengzhou University. The patients/participants provided their written informed consent to participate in this study.

## Author contributions

YMA, YMI, JW, and JZ: conceptualization. YMA, WM, DK, HF, and BZ: data curation. YMA, HF, and BZ: formal analysis. YMA and JW: funding acquisition. YMA, WM, DK, HF, and BZ: investigation. YMA and YMI: methodology. JW, and JZ: project administration. YMA and JW: writing and review editing. All authors contributed to the article and approved the submitted version.

## Funding

This work was supported by the Collaborative Innovation Key Project of Zhengzhou (No. 20XTZX05015), Key Scientific and Technological Projects in Henan Province (No. 212102310817), and National Natural Science Foundation of China (No. 72204226).

## Conflict of interest

The authors declare that the research was conducted in the absence of any commercial or financial relationships that could be construed as a potential conflict of interest.

## Publisher’s note

All claims expressed in this article are solely those of the authors and do not necessarily represent those of their affiliated organizations, or those of the publisher, the editors and the reviewers. Any product that may be evaluated in this article, or claim that may be made by its manufacturer, is not guaranteed or endorsed by the publisher.
