# Supplementary material for: Fear of COVID-19 among cancer patients in Henan Province, Central China: causes, results, and coping factors
Source: Front Psychol. 2023 Jun 16;14:1122894. doi: 10.3389/fpsyg.2023.1122894 (PMC10312372; doi:10.3389/fpsyg.2023.1122894)
Supplement: Supplementary file 1 [file Data_Sheet_1.docx]

**Appendix 1 Data collection and explanatory variables.**

# Section 1. Personal information questionnaire

# I. General Information

| 1. What's your gender？ A. male B. female |
| --- |
| 1. How old are you？ Age |
| 1. What is your marital status?   A. Unmarried B. Married C. Divorced D. Death of a spouse E. Other |
| 1. What is your education level?   A. Illiteracy B. Primary and below C. Junior high school D. Senior High School (Technical secondary school) E. University and above |
| 1. What is your occupation type？   A. Workers in transportation, construction, production, manufacturing and other industries  B. Personnel in agriculture, forestry, animal husbandry, fishing, water conservancy and other industries (farmers, fishermen, etc.)  C. Business or service workers (self-employed, service workers, etc.)  D. Students  E. Professional and technical personnel (medical workers, accountants, teachers, scientific researchers, etc.)  F. Persons in charge of state organs, party and mass organizations, enterprises and institutions  F. Retire/leave  G. Unfixed occupation  H. Other |
| 1. When did you diagnose the cancer disease? _____year_____mouth |
| 1. What is the location of your confirmed cancer disease? 2. head and neck thyroid B. nasopharynx C. oral cavity D. breast E. esophagus F. stomach   G. lung H. liver I. pancreas J. gallbladder K. colorectal L. uterus M. ovary N. prostate O. bone P. blood Q. lymph R. brain S. skin or soft tissue T. other |
| 1. Whether the cancer is metastatic? 2. Yes B. No |
| 1. What is your days of hospitalization? days |

# II． Quality of life Assessment (EQ-5D)

| 1. Do you have any difficulty getting around today？   A. No difficulty B. A little bit difficult C. Moderate difficulty D. Be in serious difficulty E. Unable to get around |
| --- |
| 1. Did you have trouble doing everyday activities like washing or dressing today？   A. No difficulty B. A little bit difficult C. Moderate difficulty D. Be in serious difficulty E. Unable to bathe or dress themselves |
| 1. Do you have any difficulty in your work, study, housework, leisure activities today？   A. No difficulty B. A little bit difficult C. Moderate difficulty D. Be in serious difficulty E. Inability to carry out daily activities |
| 1. Do you feel any pain or discomfort today？   A. No pain B. A little bit sore C. Moderate pain or discomfort D. Severe pain or discomfort E. Very severe pain or discomfort |
| 1. Are you feeling anxious or depressed today？   A. No anxiety or depression B. A little anxiety or depression C. Moderate anxiety or depression D. Have severe anxiety or depression E. Very severe anxiety or depression |
| 1. If 0 is the worst and 100 is the best, how would you rate your health today？   \|-----\|—---\|—---\|—---\|—---\|—---\|—---\|—---\|—---\|—---\|  0 10 20 30 40 50 60 70 80 90 100  Please write the number you marked on the scale in the space below.  Your health today = points |

**III. Awareness and behavior during the COVID-19 pandemic**

| 1. Do you think the COVID-19 pandemic is serious？   A. Very serious B. Serious C. General D. Not too serious E. Nothing serious at all F. Unclear |
| --- |
| 1. Is the COVID-19 outbreak serious in your place of residence？   A. Very serious B. Serious C. General D. Not too serious E. Nothing serious at all F. Unclear |
| 1. In the future, do you think the COVID-19 epidemic will continue or rebound in your place？   A. Highly possible B. Possible C. Not sure D. Impossible E. Absolutely impossible F. Unclear |
| 1. As a cancer patient, do you think you have a higher risk of COVID-19 infection than other people?   A. Highly possible B. Possible C. Not sure D. Impossible E. Absolutely impossible |
| 1. As a cancer patient, do you think that the possibility of cure after infection with COVID-19 is less than that of others?   A. Highly possible B. Possible C. Not sure D. Impossible E. Absolutely impossible |
| 1. Has the emergence of COVID-19 delayed your progress of disease diagnosis and treatment?   A. Highly agree B. Agree C. Not sure D. Disagree E. Absolutely disagree |
| 1. You are very afraid of COVID-19   A. Highly agree B. Agree C. Not sure D. Disagree E. Absolutely disagree |
| 1. The thought of COVID-19 makes you feel uncomfortable   A. Highly agree B. Agree C. Not sure D. Disagree E. Absolutely disagree |
| 1. When you think that you may get COVID-19, your hands will sweat   A. Highly agree B. Agree C. Not sure D. Disagree E. Absolutely disagree |
| 1. Your heart beats faster when you think you might get COVID-19   A. Highly agree B. Agree C. Not sure D. Disagree E. Absolutely disagree |
| 1. You are very afraid that your life will be affected by COVID-19   A. Highly agree B. Agree C. Not sure D. Disagree E. Absolutely disagree |
| 1. When you see or hear news about COVID-19, you become nervous   A. Highly agree B. Agree C. Not sure D. Disagree E. Absolutely disagree |
| 1. You can't sleep because you are worried that you will get COVID-19   A. Highly agree B. Agree C. Not sure D. Disagree E. Absolutely disagree |
| 1. Do you wash your hands more often than before COVID-19？   A. Significantly increased B. Significantly increased C. No significant change D. Decreased E. Significantly decreased |
| 1. Do you use hand sanitizer (soap) more often than before COVID-19？   A. Significantly increased B. Significantly increased C. No significant change D. Decreased E. Significantly decreased |
| 1. Are you wearing masks more often than you did before COVID-19？   A. Significantly increased B. Significantly increased C. No significant change D. Decreased E. Significantly decreased |
| 1. Do you participate in gathering activities more frequently than before COVID-19？   A. Significantly increased B. Significantly increased C. No significant change D. Decreased E. Significantly decreased |
| 1. Have you consciously increased social distancing (distance of more than 1m between people) compared to pre-COVID-19？   A. Always B. Often C. Sometimes D. Little E. Never |
| 1. Do you feel lonely when you are in hospital during the COVID-19 pandemic?   A. Always B. Often C. Sometimes D. Little E. Never |
| 1. Do you suffer from economic crisis due to the COVID-19 pandemic?   A. Highly agree B. Agree C. Not sure D. Disagree E. Absolutely disagree |
| 1. I received psychological counseling during the COVID-19 pandemic.   A. Highly agree B. Agree C. Not sure D. Disagree E. Absolutely disagree |
| 22. Do you exercise during the COVID-19 pandemic?  A. Less or never B. General C. Always |

**IV. Awareness and Behavior of COVID-19 vaccine**

| 1. Do you want to be vaccinated against COVID-19？   A. Yes and already vaccinated B. Yes, but not at that time C. Hesitation and delay D. Those refusing E. Refuse vaccination and convince people around you not to |
| --- |
| 1. In the future, do you think it is necessary for all staff to have regular vaccination against COVID-19？   A. Strongly necessary B. Be necessary C. Not sure D. Not necessary E. Totally unnecessary |
| 1. As a cancer patient, I am worried that the COVID-19 vaccine will affect my health   A. Highly agree B. Agree C. Not sure D. Disagree E. Absolutely disagree |
| 1. As a cancer patient, I am worried that the injection of COVID-19 vaccine will affect my disease treatment process   A. Highly agree B. Agree C. Not sure D. Disagree E. Absolutely disagree |
| 1. Injection of COVID-19 vaccine is only effective for the general population and may not be effective for cancer patients   A. Highly agree B. Agree C. Not sure D. Disagree E. Absolutely disagree |
| 1. Injection of covid-19 vaccine may not be safe for cancer patients   A. Highly agree B. Agree C. Not sure D. Disagree E. Absolutely disagree |
| 7. I can not obtain personalized information of COVID-19 vaccination  A. Highly agree B. Agree C. Not sure D. Disagree E. Absolutely disagree |
